# Herpes virus entry mediator signaling blockade produces mortality in neonatal sepsis through induced cardiac dysfunction

**DOI:** 10.3389/fimmu.2024.1365174

**Published:** 2024-05-07

**Authors:** Michelle E. Wakeley, Naomi-Liza Denning, Jihong Jiang, Monique E. De Paepe, Chun-Shiang Chung, Ping Wang, Alfred Ayala

**Affiliations:** ^1^ Division of Surgical Research, Department of Surgery, Brown University, Rhode Island Hospital, Providence, RI, United States; ^2^ Center for Immunology and Inflammation, The Feinstein Institutes for Medical Research, Manhasset, NY, United States; ^3^ Department of Surgery, Donald and Barbara Zucker School of Medicine at Hofstra/Northwell, Hempstead, NY, United States; ^4^ Department of Anesthesiology, Shanghai General Hospital, Shanghai Jiao Tong University School of Medicine, Shanghai, China; ^5^ Department of Pathology, Women and Infants Hospital, Providence, RI, United States

**Keywords:** HVEM, neonatal sepsis, immune dysfunction, septic cardiomyopathy, mouse

## Abstract

**Introduction:**

Sepsis remains a major source of morbidity and mortality in neonates, and characterization of immune regulation in the neonatal septic response remains limited. HVEM is a checkpoint regulator which can both stimulate or inhibit immune responses and demonstrates altered expression after sepsis. We hypothesized that signaling via HVEM would be essential for the neonatal response to sepsis, and that therefore blockade of this pathway would improve survival to septic challenge.

**Methods:**

To explore this, neonatal mice were treated with cecal slurry (CS), CS with Anti-HVEM antibody (CS-Ab) or CS with isotype (CS-IT) and followed for 7-day survival. Mice from all treatment groups had thymus, lung, kidney and peritoneal fluid harvested, weighed, and stained for histologic evaluation, and changes in cardiac function were assessed with echocardiography.

**Results:**

Mortality was significantly higher for CS-Ab mice (72.2%) than for CS-IT mice (22.2%). CS resulted in dysregulated alveolar remodeling, but CS-Ab lungs demonstrated significantly less dysfunctional alveolar remodeling than CS alone (MCL 121.0 CS vs. 87.6 CS-Ab), as well as increased renal tubular vacuolization. No morphologic differences in alveolar septation or thymic karyorrhexis were found between CS-Ab and CS-IT. CS-Ab pups exhibited a marked decrease in heart rate (390.3 Sh vs. 342.1 CS-Ab), stroke volume (13.08 CS-IT vs. 8.83 CS-Ab) and ultimately cardiac output (4.90 Sh vs. 3.02 CS-Ab) as well as a significant increase in ejection fraction (73.74 Sh vs. 83.75 CS-Ab) and cardiac strain (40.74 Sh vs. 51.16 CS-Ab) as compared to CS-IT or Sham animals.

**Discussion:**

While receptor ligation of aspects of HVEM signaling, via antibody blockade, appears to mitigate aspects of lung injury and thymic involution, stimulatory signaling via HVEM still seems to be necessary for vascular and hemodynamic resilience and overall neonatal mouse survival in response to this experimental polymicrobial septic insult. This dissonance in the activity of anti-HVEM neutralizing antibody in neonatal animals speaks to the differences in how septic cardiac dysfunction should be considered and approached in the neonatal population.

## 1 Introduction

Sepsis, a life-threatening organ dysfunction caused by a dysregulated host response to infection, remains an immense clinical challenge responsible for greater than 200,000 patient deaths annually in the United States at an estimated cost of $20 billion (1, 2). The dysregulated host response to infection seen in sepsis is characterized by circulating immune cell influx into distal organs leading to organ failure (3). While work to understand the role of this dysregulated immune response in adult septic outcomes has generated several promising therapeutic targets, little has been done to translate this understanding to the neonatal population (4). This is important as not only is this a vulnerable population for which clinical access as well as animal modeling is more limited than the adult, but the immune system of the neonate is not yet fully developed, and differences would be anticipated in its response to pathogenic challenges (5). Thus, characterization of immune regulation in the neonatal response to septic insult remains an important goal.

Sepsis in adult patients has consistently been demonstrated to produce immune deficiency, which is linked to secondary infections, organ dysfunction and, ultimately, an increased risk of death (6, 7). Early mortality in adult sepsis is most frequently secondary to cardiac dysfunction, while late mortality is most frequently attributed to secondary infection (8–10). Individuals with cardiac dysfunction have a two-fold increase in mortality after sepsis, while those developing secondary infections have worse outcomes than counterparts when matched for injury severity and age (8–10). Mortality after sepsis in neonates is also attributed to organ failure, but more specifically to respiratory distress and cardiac dysfunction. Research exploring the developing immune systems role in this response describe the neonatal reliance on an immature innate immune response, characterized by an overly robust pro-inflammatory response with rapid production of IL-6, IL-8 and TNF-α in higher concentrations than seen in adults, followed by an immature anti-inflammatory response with low IL-10 production (5). Cardiac dysfunction in adults is characterized by a hyperdynamic phase with, decreased ejection fraction and increased cardiac index, resulting from a decreased systemic vascular resistance (11). Advanced studies of coronary artery oxygenation have demonstrated these effects to not be directly related to ischemia (9). Pediatric cardiac dysfunction is instead characterized by a non-hyperdynamic phase with a decreased cardiac output and an increased systemic vascular resistance, and some of this is due to their alteration in cardiac structure where an increase in the type I to type III collagen ratio results in increased cardiac rigidity (5, 9).

The immune response to any stimulus is informed and directed by checkpoint regulators. Checkpoint regulators are a group of transmembrane protein receptors which produce a second ‘licensing’ signal following T-cell receptor stimulation by an MHC class I or II protein bound with antigen in order to instruct the cell on how to respond to the antigenic stimulus (12). When an antigen is bound by a T-cell receptor (TCR), simultaneous engagement of a checkpoint regulator results in a signal to the immune cell, which may be stimulatory or inhibitory. Stimulation results in immune activation, ultimately resulting in a humoral or cell-mediated immune response (13). Inhibitory signals can result in anergic immune cells not able to respond to further stimulation (14). Multiple checkpoint regulators exist, including the well characterized Programmed cell death receptor 1 (PD-1), Cytotoxic T-lymphocyte Associated protein 4 (CTLA-4), and V-domain Ig suppressor of T-cell activation (VISTA) (15, 16). Herpes Virus Entry Mediator, or HVEM, is another such regulator possessing several unique characteristics that make it an interesting potential therapeutic target (17).

HVEM is a tumor necrosis factor receptor superfamily (TNFRSF) member type 1 transmembrane checkpoint regulator. Discovered through its interaction with Herpes Simplex Virus, HVEM acts to stimulate or inhibit T-cell activation dependent on multiple environmental cues (17, 18). HVEM is expressed on a variety of immune cell subsets and tissues including T-cells, B-cells, and Natural Killer (NK) cells, as well as spleen, lung, liver and kidney (17, 19). HVEM has 5 described ligands, both within and outside of the TNF family including B and T Lymphocyte Attenuator protein (BTLA), CD160, LIGHT, lymphotoxin alpha (LTα), and Herpes Simplex Virus 1. HVEM possesses four cystine rich binding domains, allowing it to interact with multiple ligands simultaneously (20). HVEM can behave as a bidirectional switch, generating a stimulatory immune response when binding LIGHT or LTα, but an inhibitory signal when interacting with BTLA or CD160 (21, 22). Further, HVEM can exist in an inert form when co-expressed in its cis confirmation with BTLA, protecting naïve T-cells from responding to environmental signals (23). Altered expression of HVEM and its ligands has been demonstrated in critically ill septic adult patients, and in murine sepsis models (24–26).

Many checkpoint regulators play key roles in mediating responses to septic challenge. Deletion of both PD-1, and its primary ligand PDL-1, have both been independently demonstrated to convey survival benefit in adult mice subjected to the cecal ligation and puncture (CLP) model (27, 28). Specifically within neonates, it has been demonstrated that PD-1 deletion similarly conveys a survival benefit without effect on the intraperitoneal bacterial burden using a murine cecal slurry (CS) model (29). HVEM has been demonstrated to play an important role in the immune response to septic challenges in adult models, with Shui et al. demonstrating the HVEM : CD160 interaction to be integral to maintaining host defenses at mucosal barriers in murine models of intestinal and respiratory infections (26). Further, in an adult mouse model of indirect acute lung injury intratracheal HVEM siRNA administration was shown to have a protective effect on lung histology and convey and early survival deficit comparted with controls (30). Northern blot murine tissue surveys demonstrate the highest level of expression in adult spleen and peripheral leukocytes; however, in neonates, HVEM was most heavily expressed in lung and kidney (19). With this in mind, we hypothesized that signaling via HVEM is essential for the neonatal response to intraabdominal sepsis, and sought to characterize its role via intraperitoneal blockade of the stimulatory domains of HVEM in the setting of a septic insult.

## 2 Materials and methods

### 2.1 Mice

Wild type (WT) C57BL/6 neonatal (5-7 days old) mice were bred in the Rhode Island Hospital-Central Research Facilities. Pups were housed with parents throughout the duration of the studies and weaned on a standard schedule. All protocols were conducted according to the National Institutes of Health guide for animal care and use and were approved by the Lifespan-Rhode Island Hospital Institutional animal care and use committee (approval number: 0054-18 and 5054-21). Animals were fed standard mouse chow ad libitum, housed in ventilated racks, and kept under standard environmental conditions (12-h:12-h light/dark cycle, 68–72°F, 30%–70% humidity).

### 2.2 Cecal slurry model

Intraabdominal sepsis was created using the previously described cecal slurry (CS) model (29, 31). Briefly, cecal contents were harvested from an C57BL/6 adult male mouse, purchased through Jackson Laboratories, after CO_2_ asphyxiation, then dissolved in dextrose solution to create a slurry of 80 mg/mL concentration. For all studies administering CS, pups were weighed and treated with LD_70_ dose (1.3mg/g BW) of the dextrose cecal slurry intraperitoneally via single injection. After treatment, animals were monitored every 6 hours for 48 hours, then every 12 hours thereafter for survival studies. Sepsis modeling was designed to embody the MQTiPPS principles whenever possible (32). For studies with predetermined end points, animals were euthanized via CO_2_ asphyxiation followed by decapitation. All animal work was conducted in accordance with the Animal Welfare Act and National Institute of Health (NIH) guidelines for animal care and use, and protocols were approved by the Institutional Animal Care and Use Committee of Rhode Island Hospital (AWC# 5064-18 and 5054-21).

### 2.3 Survival studies

WT neonatal littermates were randomized into a total of 8 treatment groups, summarized in **Table 1**, including Naïve (N, N=4) pups, and pups receiving an intraperitoneal weight based dose of 0.9% saline, or Sham (Sh, N=4), cecal slurry alone (CS, N=28), CS with 20μL Anti-HVEM antibody (~4 mg protein/kg body weight) to the LIGHT stimulatory binding domain (CS-Ab, N=18) (ThermoFisher Scientific, CD270 functional grade monoclonal antibody, LH-1 clone, #16-5962-85), CS with 20μL Armenian hamster IgG isotype (~4 mg protein/kg body weight) (CS-IT, N=18) (eBiosciences, #14-4888-85), CS with 20μL 0.9% normal saline (CS-NS, N=19), 20μL Anti-HVEM antibody to the LIGHT stimulatory binding domain alone (Ab,N=4), or 20μL Anti-HVEM antibody diluted in a weight based volume of normal saline (dAb, N=4). Antibody dosing was selected based on previously published work in adult models (33). Antibody dosing was exclusively administered at the time of CS treatment; no additional dosing was provided. Given all litters did not include 8 pups, every experimental litter was ensured to include control animals (N, Sh, CS, Ab, or dAb treated mice). Pups from each treatment group underwent a 7-day survival study. Following intraabdominal injection pups were evaluated every 6hrs for the first 48hrs, then every 12hrs for the remainder of the study. No antibiotics, pain medications, or additional resuscitative fluids were administered. Results were analyzed using a log-rank Kaplan Meier analysis.

**Table 1:** Experimental treatment groups.

Treatment	Label	Technique	N
Naïve	N	No treatment	5
Sham	Sh	Intraperitoneal weight-based dose 0.9% saline	4
Cecal Slurry	CS	Cecal slurry alone	28
Cecal Slurry with Isotype	CS-IT	CS with 20μL Armenian hamster IgG isotype	18
Cecal Slurry with Anti-HVEM Antibody	CS-Ab	CS with 20μL Anti-HVEM antibody to the LIGHT stimulatory binding domain	18
Cecal Slurry with Normal Saline	CS-NS	CS with 20μL 0.9% normal saline	19
Anti-HVEM Antibody	Ab	20μL Anti-HVEM antibody to the LIGHT stimulatory binding domain	4
Diluted Anti-HVEM Antibody	dAb	20μL Anti-HVEM antibody diluted in a weight-based volume of normal saline	4

Summary of all treatment groups included in study design, including details of treatment technique and number of pups included in survival studies for each treatment.

### 2.4 Organ weights

Twelve hrs after treatment, thymus, lung, liver, spleen and kidney samples from naïve mice (N), Sham (Sh), CS, CS-Ab or CS-IT treated mice were collected following euthanasia via decapitation. Pups were weighed at the time of euthanasia and organs were weighed immediately after collection (wet weight). All weights are reported as a ratio of the organ weight in milligrams to the weight of the pup in grams (**Table 2**). Samples were then formalin fixed for histologic evaluation.

**Table 2:** Wet tissue weights as a ratio with pup weight.

Treatment	Pup Weight	Thymus	Lung	Liver	Spleen	Kidney*
Naïve (N=8)	3.51 +/- 0.65	6.41 +/- 1.2	16.33 +/- 1.61	29.27 +/- 5.09	6.25 +/- 1.42	11.25 +/- 2.47
Cecal Slurry (N=5)	3.83 +/- 0.08	2.81 +/- 1.11	12.78 +/- 2.55	17.08 +/- 3.03	8.17 +/- 1.75	7.67 +/- 1.67
CS-IT(N=5)	3.23 +/- 0.38	4.42 +/- 1.53	14.23 +/- 1.73	23.42 +/- 4.13	9.58 +/- 2.62	8.75 +/- 0.67
CS-Ab (N=5)	3.56 +/- 0.26	5.4 +/- 1.53	11.4 +/- 1.5	23.18 +/- 4.67	8.32 +/- 1.59	5.14 +/- 0.52

Treatment groups contained between 5 and 8 pups, pup weight is provided as a group average in grams with the SEM, tissue weights are reported as a ratio of tissue weight in milligrams per pup weight in grams along with SEM. * denotes significant difference between CS-IT and CS-Ab groups, p<0.05.

### 2.5 Histology

Thymus, lung, liver, spleen and kidney formalin fixed samples collected from Sh, CS, CS-Ab or CS-IT treated pups 12 hrs after treatment as well as from Naïve mice were paraffin embedded for sectioning. Sections were either stained with hematoxylin and eosin (H&E), underwent immunohistochemical analysis for HVEM expression (ThermoFisher Scientific, TNFRSF14 Polyclonal antibody, #PA5-20237), or were assessed for apoptotic changes with active Caspase 3 (Clone CM1, BD Biosciences).

H&E-stained lung samples were evaluated by computer assisted morphometric analysis for evidence of age-appropriate alveolar remodeling via mean chord length (MCL). MCL was calculated by superimposing randomly oriented parallel line arrays over a high-powered field (40x magnification) of air exchanging lung parenchyma, then determining the distance between airspace walls, as previously described (34). This process was repeated across 10 random fields per lung then averaged to generate the MCL score, in μm. MCL data is reported as mean distance in μm for an N=4 in each treatment group ± SEM.

Based on prior reports of lymphoid apoptosis as a characteristic result of histologic murine septic tissue injury, H&E stained thymus samples were evaluated for lymphoid apoptosis, or karyorrhexis, utilizing a karyorrhectic index (KI) (35). A karyorrhectic foci was defined as an area containing 3 or more nuclear fragments, and the karyorrhectic index was calculated based on the number of foci identified in 10 random high-powered fields (40x magnification) per tissue sample. KI data is reported as mean for an N=4 in each treatment group ± SEM.

H&E stained kidney samples (N=4 per treatment group) were evaluated for evidence of vacuolar degeneration, tubular epithelial swelling, and desquamation, based on prior descriptions of these characteristic murine indicators of kidney injury (35–37). Images were first acquired at 20x magnification capturing the cortex, then images were magnified to 40x and evaluated for general morphology and presence and extent of vacuolar degeneration and tubular epithelial swelling. This was repeated across 2-3 areas of each tissue sample.

Thymic samples (N=5 per treatment group) were evaluated for HVEM expression utilizing a commercially available polyclonal antibody using the manufacturers protocol. Images were acquired at 40x magnification within the cortical zones for each tissue sample and compared grossly for stain intensity. Thymus samples (N=5 per treatment group) were evaluated for active Caspase 3 according to manufacturer instructions, and additionally underwent immunohistochemical analysis for HVEM expression. Images of 6 random low-powered fields per sample were acquired at 4x magnification and analyzed for stain intensity using ImageJ. Of note, all histologic samples mentioned above were reviewed by a single neonatal pathologist member of the research team (MED) in a blinded fashion utilizing ImageJ software. All results were compared using a Mann-Whittney U test.

### 2.6 Peritoneal culture analysis

Normal saline was lavaged in the peritoneal cavity of pups 12hrs after treatment with CS, CS-Ab, CS-IT, CS-NS, AB or dAb as well as in Naïve mice (N). Samples were collected at the time of tissue harvest using sterile technique, aliquoted, and cultured for 24 hrs on blood agar plates at 37°C then counted for colony forming units (CFUs). Results are reported as Log_10_ CFU/100μL peritoneal fluid.

### 2.7 Echocardiography

Twenty-four hrs after treatment, either Sh treatment with normal saline, or sepsis induction with CS-Ab or CS-IT, transthoracic echocardiography was used to assess cardiac function in neonatal mice as described by Denning et al. (38) Echocardiography was completed using a 40 MHz center frequency transducer and a Vevo^®^3100 Imaging System (Fujifilm VisualSonics, Toronto, ON, Canada). Sedation was induced with 2.5% isoflurane, and subsequently was maintained with 0.5–1% isoflurane throughout the echocardiogram. Mice were maintained on a heated table throughout the echocardiogram. Parasternal long axis views were taken in B and M modes, with B mode imaging providing two-dimensional views of the heart and M mode imaging depicting one ultrasound line, chosen from the two-dimensional image, over time (39). VevoLab (Fujifilm VisualSonics) software was used to determine cardiac parameters. VevoStrain (Fujifilm VisualSonics) software was used to measure myocardial strain and strain rate using speckle-tracking echocardiography (40).

### 2.8 Cytokine arrays

Twenty-four hrs after treatment plasma, thymus, lung, and kidney samples from naïve mice (N), and Sham (Sh), CS, CS-Ab, CS-IT, CS-NS, Ab, or dAb treated mice were collected following euthanasia and stored at -80°C until analysis. Blood samples were collected with sterile technique at the time of decapitation, plasma was subsequently isolated via centrifugation of whole blood at 10,000 rpm, and supernatant (plasma) was collected and stored at -80°C. Plasma samples were analyzed for circulating cytokine profiles according to manufacturer instructions using a commercially available cytokine bead array: LEGENDplex Mouse Inflammation Panel (13-plex) with a V-bottom plate (BioLegend, cat#552364). Serum samples from murine subjects were prepared for analysis per manufacturer’s protocol and as previously described by our lab (41). Lung, thymus, and kidney samples were dissociated into cell lysates and prepared for cytokine bead array according to manufacturer protocols. MACSQuant Analyzer 10 (Miltenyi Biotec) was utilized for analysis of all multiplex experiments. Results were analyzed with LEGENDplex software suite (BioLegend). Differences among multiple groups were established with non-parametric Kruskal-Wallis test followed by post-hoc Dunns test using a Bonnferroni correction.

### 2.9 Statistical analysis

Data are expressed as mean ± SEM. Survival study data was analyzed using a log-rank Kaplan Meier analysis. Multi-group comparisons were performed using Kruskal-Wallis test followed by post-hoc Dunns test using a Bonnferroni correction or a Mann-Whittney U test for simple 2-way comparison. Statistical analysis was undertaken using GraphPad Prism version 8.4.3 for Windows (GraphPad Software, San Diego, California USA, www.graphpad.com). Alpha was set to 0.05.

## 3 Results

### 3.1 Neonatal mortality is increased after septic insult in the setting of HVEM signaling blockade, occurring despite the resuscitative benefit of additional volume administration

**Figure 1:**
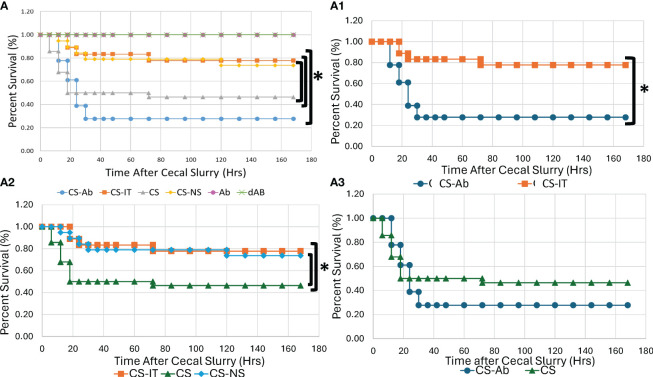
Neonatal murine survival after sepsis with intraperitoneal HVEM blockade depicted in Kaplan-Meier curves. **(A)** All Treatment groups. **(A1)** Intraperitoneal HVEM blockade with CS produces a significant survival deficit compared to isotype control, with 5/18 for CS-Ab vs. 14/18 CS-IT groups (p=0.003) **(A2)** CS-IT treated pups survival demonstrated no significant difference from survival of pups treated with CS-NS (14/18 CS-IT vs. 14/19 CS-NS, p=0.949), yet survived significantly better than pups treated with CS alone (14/18 CS-IT vs. 13/28 CS, p=0.035) **(A3)** CS-Ab treatment produced slightly worse survival than CS alone (5/18 CS-Ab vs. 13/28 CS, p=0.327) and the text box labels under **Figure 1A1** has been shifted revealing something that was covered with a white box on the version I sent in, the text boxes should be moved to the left 2-3 tabs.

Mortality was significantly higher for CS-Ab mice than for CS-IT mice, with 5 of 18 CS-Ab treated mice survived versus 14 of 18 CS-IT treated mice over a 7-days (p=0.003), depicted in **Figure 1A**, **A1**. Within the CS-Ab group, mortality was noted to be rapid, with 84% of deaths occurring by the 24 hr time point. When evaluating CS-IT mice compared to CS treatment alone or CS-NS treatment (**Figure 1A2**) CS-IT mice survived significantly better than CS alone with 14/18 CS-IT pups surviving to 7 days compared to 13/28 CS pups (p=0.035). However, CS-IT treated pups survival demonstrated no significant difference from survival of pups treated with CS-NS (14/18 CS-IT vs. 14/19 CS-NS, p=0.949). CS-Ab mice demonstrate slightly worse survival than pups treated with CS alone, as shown in **Figure 1A3** (p=0.327). Anti-HVEM antibody administration independently, both alone (Ab) in diluted in normal saline (dAb), generated no effect on lethality (**Supplementary Figure 1**). Finally, consistent with results of prior work within the CS model by our lab, there was no mortality demonstrated for naïve or Sham treatments (**Supplementary Figure 1**) (29, 42).

### 3.2 Dysregulated alveolar remodeling associated with CS model and attenuated by HVEM blockade

**Figure 2:**
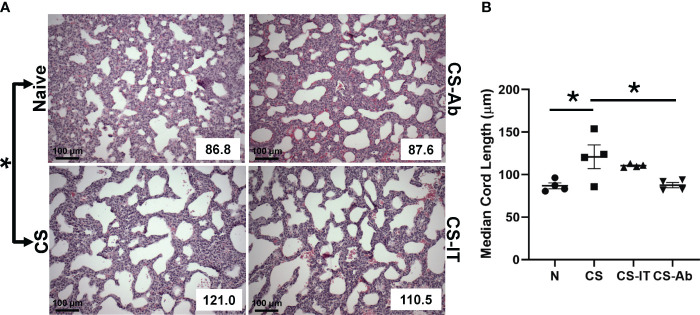
Dysregulated alveolar remodeling associated with CS model and attenuated by HVEM blockade: **(A)** H&E stained samples of lungs harvested from Naïve, CS, CS-IT and CS-Ab treated pups 12hrs after IP injection depicted at 40x magnification with mean chord length (MCL) reflected in the lower right corner, provided in μm. **(B)** Histogram depicting the average MCL ± SEM per group: 86.88 μm ± 3.43 N, 97.85 μm ± 9.88 Sh, 120.95 μm ± 13.94 CS, 110.53 μm ± 0.96 CS-IT, and 87.55 μm ± 3.13 CS-Ab. MCL was significantly increased in CS compared with Naïve (p<0.05), and significantly lower in CS-Ab pups than in CS alone (p<0.05). Finally, CS-Ab treated lung samples were found to have significantly shorter MCL than CS-IT treated pups (p=0.029); data is shown as dot plot with mean +/- SEM; * delineates significant differences between specific groups (bar) at p<0.05.

Histologic responses to HVEM signaling blockade in the lung were evaluated by measuring median cord length, a measure of the normal neonatal process of alveolar remodeling. Naïve lung samples demonstrated appropriate remodeling, while induction of sepsis via CS resulted in significantly dysregulated alveolar remodeling, reflected by an increase in MCL from 86.88 ± 3.43 in naïve mice to 120.95 ± 13.94 in CS treated pups (p <0.05) (**Figure 2**). Lungs from CS-Ab mice demonstrated a significant reduction in dysfunctional alveolar remodeling compared to CS alone with an MCL of 87.55 ± 3.13 in CS-Ab versus 120.95 ± 13.94 in CS treated samples (p<0.05). CS-Ab lung histology and MCL was found to be most similar to naïve lung samples with their MCL of 86.88 ± 3.43, and CS-Ab MCL was additionally found to be significantly lower than CS-IT treated samples (MCL: 87.55 ± 3.13 CS-Ab vs 110.53 ± 0.96 CS-IT, p= 0.029).

### 3.3 Sepsis induced thymic karyorrhexis, and this was not altered by CS-Ab or CS-IT treatment, while renal histology demonstrated vacuolar degeneration and mild tubular epithelial swelling

**Figure 3:**
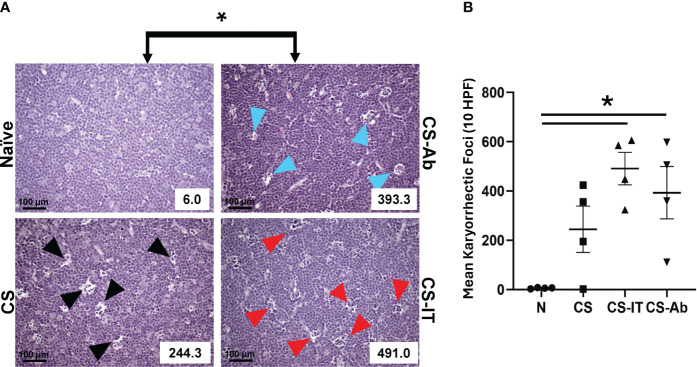
**(A)** Histologic evidence of increased thymic karyorrhexis after CS, not affected by Ab or IT treatment. H&E-stained thymic samples harvested from Naïve, CS, CS-IT and CS-Ab treated pups 12hrs after IP injection depicted at 40x magnification with karyorrhectic index (KI) reflected in the lower right corner. **(B)** Histogram of karyorrhexis indices for each treatment group. Average KI ± SEM: 6 ± 1.08 N, 244.25 ± 93.94 CS, 491 ± 65.82 CS-IT, and 393.25 ± 106.16 CS-Ab. CS-Ab and CS-IT treated pups had significantly higher KI than naïve pups (p<0.01) yet were not significantly different than KI observed in mice treated with CS alone (p>0.05); data is shown as dot plot with mean +/- SEM; * delineates significant differences between specific groups (bar) at p<0.05.

**Figure 4:**
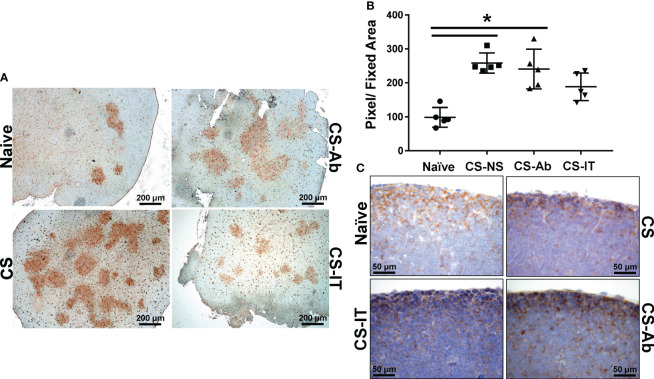
Thymic apoptosis is induced by CS administration, attenuated by IT administration. **(A)** Thymus samples harvested from Naïve, CS, CS-IT and CS-Ab treated pups 12hrs after IP injection depicted at 4x magnification following staining for caspase 3 activity. Naïve samples demonstrate mild staining, while staining is increased by CS treatment, CS-Ab samples demonstrated very similar levels of staining to CS samples, while CS-IT demonstrated mild staining. **(B)** Image densitometry plots generated by random sampling of thymic samples stained for active caspase 3 demonstrating no significant difference between CS, CS-Ab, and CS-IT treatment groups caspase 3 staining. All 3 groups treated with CS demonstrate significantly higher active caspase 3 staining than the naïve control. **(C)** Thymus samples harvested from Naïve, CS, CS-IT and CS-Ab treated pups 12hrs after IP injection depicted at 40x magnification following staining for HVEM expression with a polyclonal anti-HVEM antibody. Induction of sepsis results in grossly decreased cortical HVEM staining, which was increased back to naïve levels by CS-Ab administration; data is shown as dot plot with mean +/- SEM; significance * p<0.05.

Thymic samples demonstrated increased karyorrhexis induced by CS administration, when compared to Sham samples, 2.75 ± 1.44 Sh vs. 244.25 ± 93.94 CS (**Figures 3A, B**). CS and CS-Ab had similar increase in thymic karyorrhexis (244.25 ± 93.94 CS vs 393.25 ± 106.16 CS-Ab, p>0.05). CS-Ab samples demonstrated no significant difference when compared to CS-IT samples in terms of thymic lymphoid karyorrhexis (491 ± 65.82 CS-IT vs. 393.25 ± 106.16 CS-Ab., p>0.05). Caspase 3 staining of thymic tissue samples revealed that induction of sepsis via CS resulted in an increased amount of apoptosis within the thymus as compared with naive samples (**Figure 4A**). This response was similar in the anti-HVEM treated samples but was somewhat ameliorated in CS-IT treated pups. By densitometry, induction of sepsis via CS resulted in significantly higher levels of activated caspase 3 staining in all 3 septic groups compared to naïve, with no significant differences between CS, CS-Ab and CS-IT samples (**Figure 4B**). CS treated samples showed altered density of HVEM-immunoreactive thymocytes, especially in the subcortical zone compared to naïve (**Figure 4C**). Grossly, cortical HVEM expression decreased in CS compared to Naïve samples, yet HVEM expression was increased after CS-Ab and CS-IT treatments, more closely resembling Naïve samples.

H&E-stained renal samples (**Supplementary Figures 2A, B**) demonstrated expected apical vacuolization in Naïve samples, while CS samples demonstrated mildly increased tubular epithelial swelling with minor vacuolar degeneration, CS-IT samples with more variable findings, and, finally, CS-Ab samples demonstrated partial tubular vacuolar degeneration throughout the samples reviewed and showed mild tubular epithelial swelling. Kidney weight was significantly lower in CS-Ab than CS-IT (5.14mg/g ± 0.523 vs 8.75mg/g ± 0.665, p<0.05) (**Table 2**).

### 3.4 HVEM signaling blockade results in significant cardiac dysfunction when compared to both Sham and CS-IT treatment

**Figure 5:**
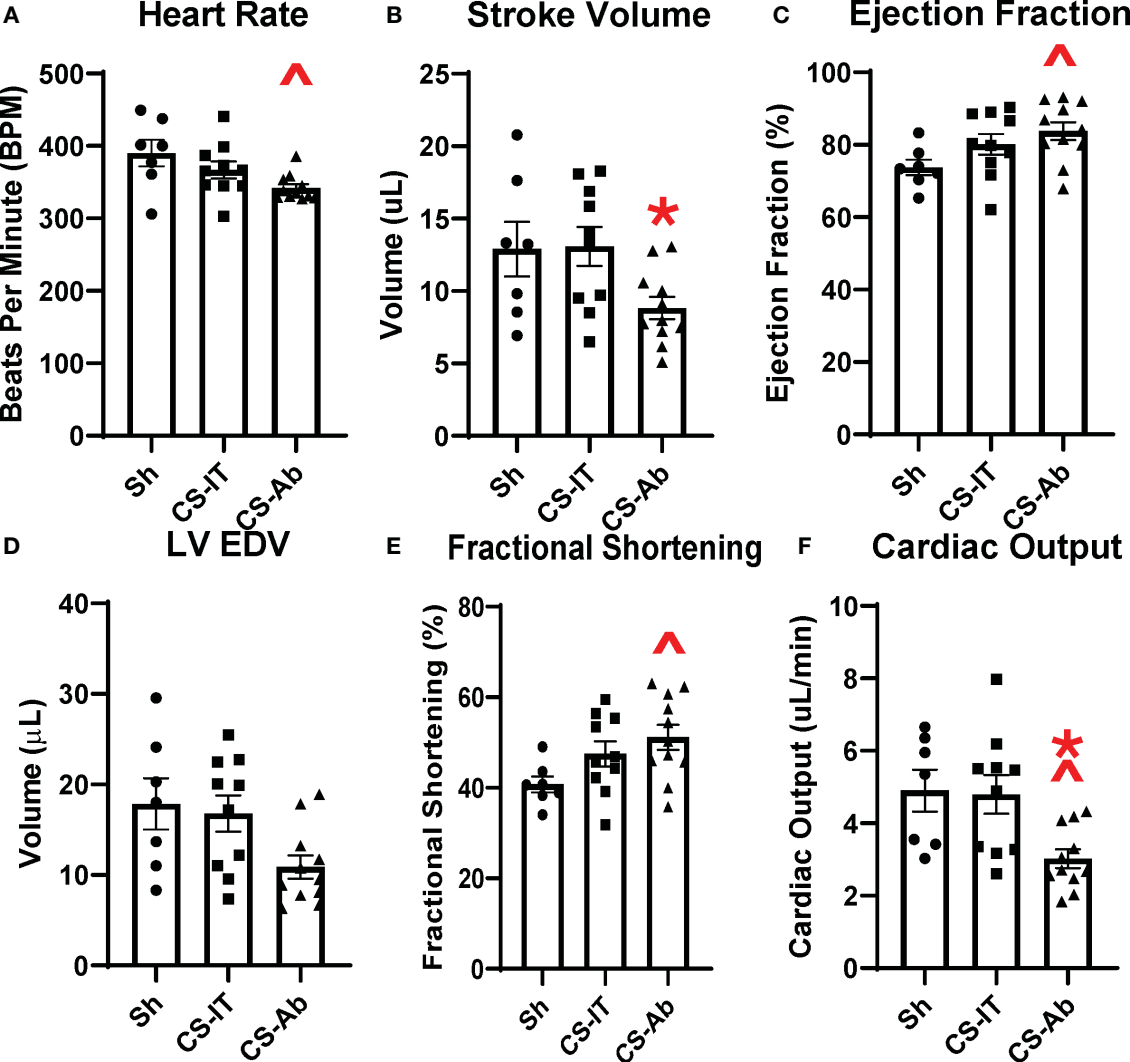
Murine echocardiography completed 24 hrs after treatment with Sh (N=7), CS-IT (N=10) or CS-Ab (N=11) **(A)** Heart rate was significantly reduced by CS-Ab treatment compared to sham control (390.3 Sh vs. 342.1 CS-Ab, p=0.020) **(B)** Stroke volume was significantly reduced 24 hrs after CS-Ab treatment compared to CS-IT (13.08 CS-IT vs. 8.83 CS-Ab; p=0.048) **(C)** CS-Ab treatment significantly increased ejection fraction compared to sham pups at 24 hrs post treatment (73.74 Sh vs. 83.75 CS-Ab; p=0.041) **(D)** Left Ventricular End Diastolic Volume was not significantly altered by treatments. **(E)** Fractional shortening was significantly increased by CS-Ab treatment compared to Sham (40.74 Sh vs. 51.16 CS-Ab; p=0.035). **(F)** Cardiac output was significantly reduced after CS-Ab treatment compared to both Sh and CS-IT treated animals. (4.90 Sh vs. 4.80 CS-IT vs. 3.02 CS-Ab; p=0.022 vs. Sh; p = 0.017 vs. CS-IT); data is shown as dot plot with mean +/- SEM; significance: ^= p<0.05 vs. Sh; *=p<0.05 vs. CS-IT.

Echocardiography 24 hrs after treatment with Sh, CS-IT and CS-Ab demonstrated that CS-Ab treatment significantly reduced heart rate in neonatal mice when compared to Sh controls (390.3 Sh vs. 342.1 CS-Ab, p=0.020), as depicted in **Figure 5A**. Similarly, CS-Ab treatment resulted in a decreased stroke volume, or volume of blood pumped out of the left ventricle in each cardiac cycle, when compared to IT controls (13.08 CS-IT vs. 8.83 CS-Ab; p=0.048), though significant variability in the stroke volume of the Sh treated mice meant no difference was appreciated between Sh and CS-IT or CS-Ab (**Figure 5B**). When looking at ejection fraction, or the percentage of blood the left ventricle pumps with each heartbeat, there was no significant difference between Sh and CS-IT treated pups (73.74 Sh vs. 80.13 CS-IT; p=0.255), however, CS-Ab treatment resulted in a significant increase in the ejection fraction compared to Sh (73.74 Sh vs. 83.75 CS-Ab; p=0.041; **Figure 5C**). CS-IT treatment closely resembled Sh treatment when left ventricular end diastolic volume was investigated, though CS-Ab treatment resulted in a trend towards reduced left ventricular end diastolic volume (LVEDV) when compared to Sh or CS-IT treatment, while Sh and CS-IT treatments resulted in nearly identical LVEDV (Sh vs. CS-IT p=0.931; Sh vs. CS-Ab p=0.057, CS-IT vs. CS-Ab p=0.077; **Figure 5D**). Antibody treatment was further associated with increased fractional shortening, a surrogate for strain, when compared to Sh pups (40.74 Sh vs. 51.16 CS-Ab; p=0.035; **Figure 5E**). Finally, it was found that CS-Ab treated pups had a reduced cardiac output compared to both sham and IT controls (4.90 Sh vs. 4.80 CS-IT vs. 3.02 CS-Ab; p=0.022 vs. Sh; p = 0.017 vs. CS-IT; **Figure 5F**).

### 3.6 Sepsis via CS induced significant differences in multiple circulating cytokine levels, including IFN-γ, TNF-α, MCP-1, IL-10 and IL-6 when compared to naïve and sham controls, however no significant differences between CS-IT or CS-Ab treatment were detected

**Figure 6:**
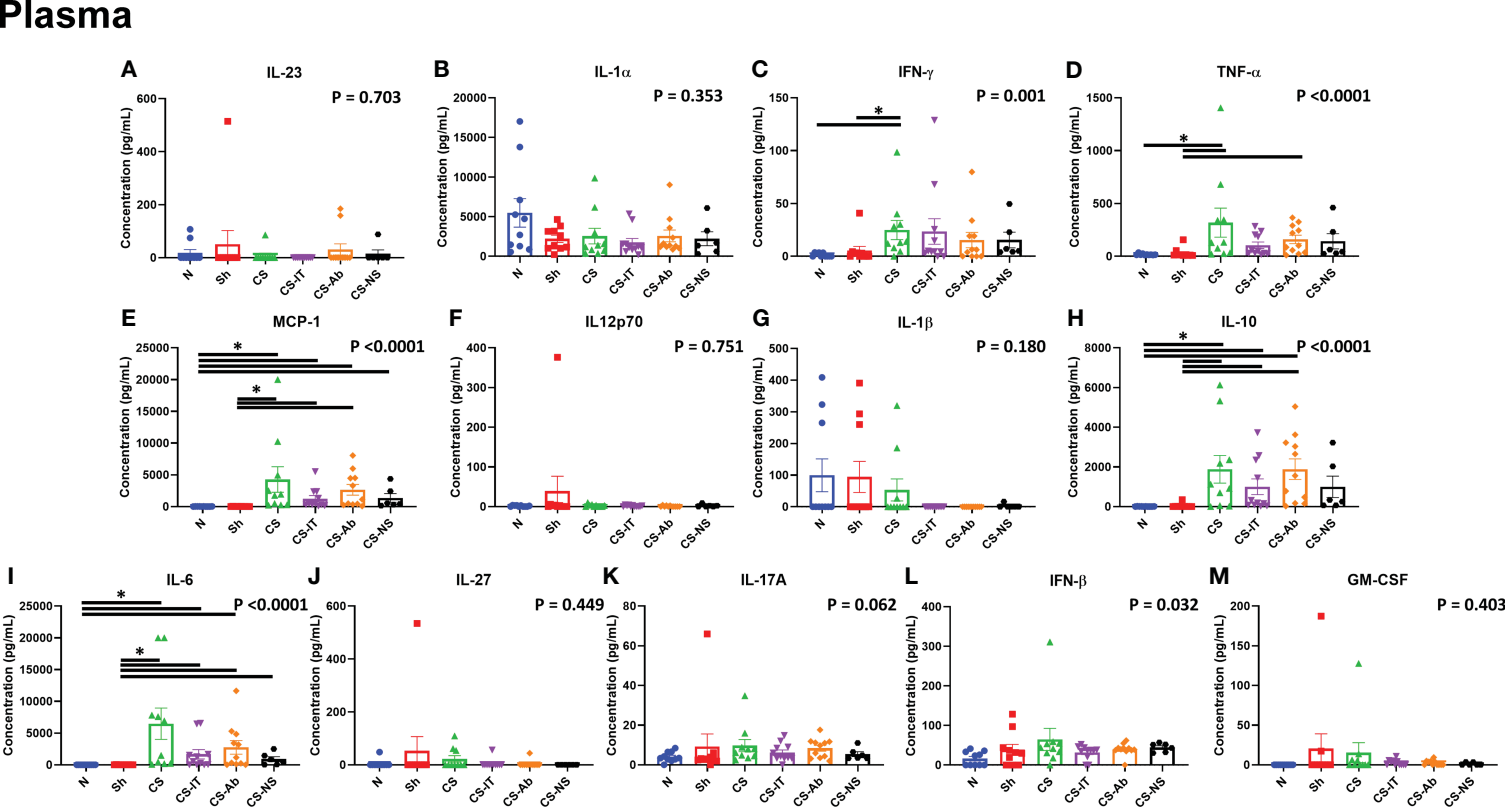
Circulating cytokine levels in neonatal mice 24 hours after treatment, mice demonstrate increases in multiple pro-inflammatory cytokines at the 24hr mark after CS treatment, though no specific differences between CS alone, CS-IT or CS-Ab treatments existed. Reported p value reflects non-parametric one-way ANOVA, remainder of indications reflect multiple comparisons test results. **(A)** IL-23 **(B)** IL-1α **(C)** IFN-γ **(D)** TNF-α **(E)** MCP-1 **(F)** IL12p70 **(G)** IL-1β **(H)** IL-10 **(I)** IL-6 **(J)** IL-27 **(K)** IL-17A **(L)** IFN-β **(M)**GM-CSF **(A–M)** Summary graphs show data dot plot and mean +/- SEM all after N, Sh, CS, CS-IT, CS-Ab, and CS-NS treatments, [N *n=10*, Sh *n=10*, CS *n=10*, CS-IT *n=11*, CS-Ab *n=11*, CS-NS *n=6*, Ab *n=6*, dAb *n=6*]; * significant differences between specific groups (bar) delineated relative to Bonferroni corrected “P” value.

The plasma cytokine array, depicted in **Figure 6**, and expanded on in **Supplementary Figures 3** and **4**, demonstrated multiple differences induced by cecal slurry treatment. IFN-γ levels varied significantly between the 8 tested treatment groups (N, Sh, CS, CS-IT, CS-Ab, CS-NS, Ab, and dAb; p=0.001; **Figure 6C**; **Supplementary Figure 3**), while it was specifically detected that sepsis without any resuscitation or antibody treatment (CS) induced significant difference compared to both naïve and sham groups (N vs. CS p=0.0046; Sh vs. CS p=0.041). TNF-α levels also varied significantly between groups (p<0.0001; **Figure 6D**). Similar to IFN-γ, CS treatment induced significant differences in TNF-α levels compared to both naïve and sham groups (N vs. CS, p=0.0426; Sh vs. CS p=0.0035). However, CS-Ab treatment also significantly increased TNF-α levels compared to Sham controls (Sh vs. CS-Ab, p = 0.014), while CS-IT treatment did not (Sh vs. CS-IT, p=0.052). MCP-1 additionally demonstrated a significant difference between the 8 treatment groups (p<0.0001), with a significant increase in levels after CS, CS-IT, and CS-Ab levels when compared to both naïve and sham pups (N vs. CS, p=0.0038; N vs. CS-IT, p=0.0352; N vs. CS-Ab, p=0.0036; Sh vs. CS, p=0.0005; Sh vs. CS-IT, p=0.0056; and Sh vs. CS-Ab, p = 0.0004) (**Figure 6E**). CS-NS treatment did not induce a significant increase in MCP-1 compared to naïve mice (N vs. CS-NS, p=0.1268), though it did compared to sham treatment (Sh vs. CS-NS, p=0.0315). No difference was detected between MCP-1 levels at 24 hrs after treatment with CS-IT and CS-Ab (CS-IT vs. CS-Ab, p>0.999). Circulating IL-10 levels were also found to be significantly different between the 8 treatment groups (p<0.0001), and similar to MCP-1 this was found to be due to significant increases after any treatment including CS (CS, CS-IT, and CS-Ab [**Supplementary Figure 4**]) excepted CS-NS, the resuscitative group, when compared to both naïve and sham pups (N vs. CS, p=0.0106; N vs. CS-IT, p=0.0139; N vs. CS-Ab, p=0.0007; Sh vs. CS, p=0.0178; Sh vs. CS-IT, p=0.0233; Sh vs. CS-Ab, p = 0.0012; N vs. CS-NS, p=0.0749; Sh vs. CS-NS, p=0.1103). Finally, circulating IL-6 levels followed a very similar pattern, with significant variability between the treatment groups (p<0.0001); related to significant increases after CS treatment groups CS, CS-IT, and CS-Ab compared to naïve and sham groups (N vs. CS p=0.0006; N vs. CS-IT, p=0.0228; N vs. CS-Ab, p=0.005; Sh vs. CS, p<0.0001; Sh vs. CS-IT, p=0.0044; Sh vs. CS-Ab, p = 0.0008). Like MCP-1, CS-NS resuscitation resulted in no significant difference compared to naïve pups (N vs. CS-NS, p=0.1107), but did induce a difference compared to sham controls (Sh vs. CS-NS, p=0.0325), and again, no difference between CS-IT and CS-Ab mice was detected (CS-IT vs. CS-Ab, p>0.999).

### 3.7 Sepsis induced renal NGal expression and tissue specific increases in inflammatory cytokines TNF-α, MCP-1, IL-10 and IL-6, however HVEM:LIGHT blockade did not alter this expression profile

**Figure 7:**
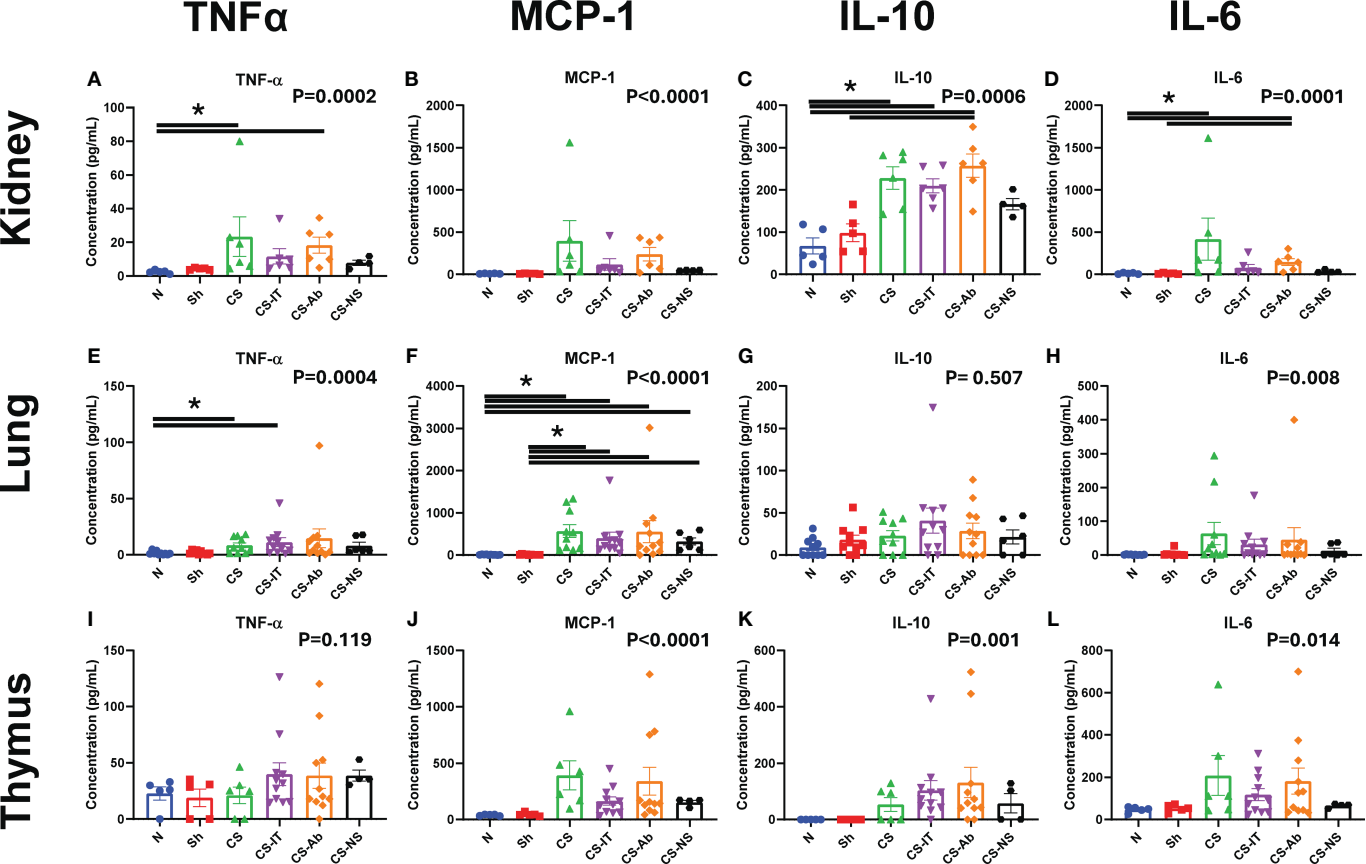
Tissue specific cytokine levels 24 hours after treatment in neonatal mice. Mice demonstrate increases in MCP-1 levels in tissue consistently after treatment, while TNFα, IL-10 and IL-6 levels are less diffusely altered. Reported p value reflects non-parametric one-way ANOVA, remainder of indications reflect multiple comparisons test results. **(A)** Kidney TNFα **(B)** Kidney MCP-1 **(C)** Kidney IL-10 **(D)** TNF-α **(E)** Lung TNFα **(F)** Lung MCP-1 **(G)** Lung IL-10 **(H)** Lung IL-6 **(I)** Thymus TNFα **(J)** Thymus MCP-1 **(K)** Thymus IL-10 **(L)** Thymus IL-6 **(A–L)** Summary graphs show data dot plot and mean +/- SEM all after N, Sh, CS, CS-IT, CS-Ab, and CS-NS treatments, [Kidney N *n=5*, Sh *n=5*, CS *n=6* CS-IT *n=6*, CS-Ab *n=6*, CS-NS *n=4;* Lung N *n=10*, Sh *n=10*, CS *n=10*, CS-IT *n=11*, CS-Ab *n=11*, CS-NS *n=6;* Thymus N *n=5*, Sh *n=5*, CS *n=6*, CS-IT *n=11*, CS-Ab *n=11*, CS-NS *n=4*]; significant differences between specific groups (bar) delineated relative to Bonferroni corrected “P” value. * p<0.05.

Western blotting using renal protein samples, depicted in **Supplementary Figure 2C**, demonstrate that NGal expression is induced by the administration of CS and maintained in both the CS-Ab and CS-IT groups. Kidney cytokine levels demonstrated the most significant changes as a result of CS treatment compared to sham and naïve control pups. First, TNF-α levels were significantly altered amongst treatment groups (p=0.0002; **Figure 7A**), with CS and CS-Ab leading to increased levels (N vs. CS, p=0.0256; N vs. CS-Ab, p=0.0062). TNF-α levels were also increased after CS-Ab treatment when compared to dAb (CS-Ab vs. dAb, p=0.0280). Kidney MCP-1 levels varied significantly between treatment groups (p<0.0001, **Figure 7B**), with specific increases in CS and CS-Ab when compared to independent antibody administration (CS vs. Ab, p=0.0291; CS-Ab vs. Ab, p=0.0196). IL-10 levels in kidney samples varied significantly between the 8 treatment groups (p=0.0006, **Figure 7C**), with CS resulting in increased IL-10 when compared to naïve, except in the setting of resuscitation with normal saline (N vs. CS, p=0.0173; N vs. CS-IT, p=0.0401; N vs. CS-Ab, p=0.0028; N vs. CS-NS, p>0.999), but only CS-Ab treatment increased IL-10 compared to sham (Sh vs. CS-Ab, p=0.0330; Sh vs. CS-NS, p>0.999). However, no difference in renal IL-10 levels between CS-IT and CS-Ab treated pups was found (CS-IT vs. CS-Ab, p>0.999). Finally, kidney IL-6 levels were significantly altered by treatment (p=0.0001, **Figure 7D**). Treatment with CS and CS-Ab resulted in significantly higher levels of IL-6 than naïve and sham, while treatment with CS-IT and CS-NS did not (N vs. CS, p=0.0126; N vs. CS-IT, p=0.1423; N vs. CS-Ab, p=0.0151; N vs. CS-NS, p=0.7793; Sh vs. CS-Ab, p = 0.0186). No difference in renal IL-6 levels between CS-IT and CS-Ab was detected (CS-IT vs. CS-Ab, p>0.999). No differences were detected between groups in the other 9 tested cytokines, as depicted in **Supplementary Figure 5**.

Lung samples also demonstrate cytokine level fluctuation. TNF-α levels varied significantly between the tested groups (p=0.0004; **Figure 7E**), with differences after CS induction by CS or CS-IT, though not by CS-Ab (N vs. CS, p=0.0408; N vs. CS-IT, p=0.0171; N vs. CS-Ab, p=0.1063). MCP-1 levels varied significantly between the 8 treatment groups (p<0.0001; **Figure 7F**), with sepsis inciting increases in CS, CS-IT, CS-Ab and CS-NS treated pups compared to naïve or sham animals (N vs. CS, p=0.0011; N vs. CS-IT, p=0.0048; N vs. CS-Ab, p=0.0216; N vs. CS-NS, p=0.0399; Sh vs. CS, p=0.0009; Sh vs. CS-IT, p=0.0039; Sh vs. CS-Ab, p = 0.0176; Sh vs. CS-NS, p=0.0338). Unlike other tissues, no significant difference in IL-10 levels were noted between the treatment groups (p=0.507; **Figure 7G**), though IL-6 levels did vary between the 8 treatment groups (p=0.008; **Figure 7H**), though no specific difference between individual groups means was detected when multiple comparisons testing was completed. No differences were detected between groups in the other 9 tested cytokines, as depicted in **Supplementary Figure 6**.

When looking at cytokine levels in the thymus, there was no significant difference in TNF-α levels between any groups (p= 0.119; **Figure 7I**). Thymus samples did however demonstrate significant variation in MCP-1 levels between the 8 tested groups (p<0.0001; **Figure 7J**). This MCP-1 variability was driven by differences between CS and CS-Ab treated groups when compared to Ab or dAb treated mice (CS vs. Ab, p=0.0182; CS vs. dAb, p=0.0049; CS-Ab vs. dAb, p=0.0241). IL-10 levels in the thymus also significantly varied amongst the 8 tested groups (p=0.001, **Figure 7K**), however no marked difference between any two individual groups was detected via multiple comparisons testing. The same was true of thymic IL-6 levels, where significant variability between the 8 tested groups (p=0.014, **Figure 7L**), but no significant difference between any two specific groups was observed. No differences were detected between groups in the other 9 tested cytokines, as depicted in **Supplementary Figure 7**.

### 3.8 Cecal Slurry treatment produced a significant increase in intraperitoneal bacterial burden; however, this was not altered by CS-Ab, CS-IT or CS-NS treatments

**Figure 8:**
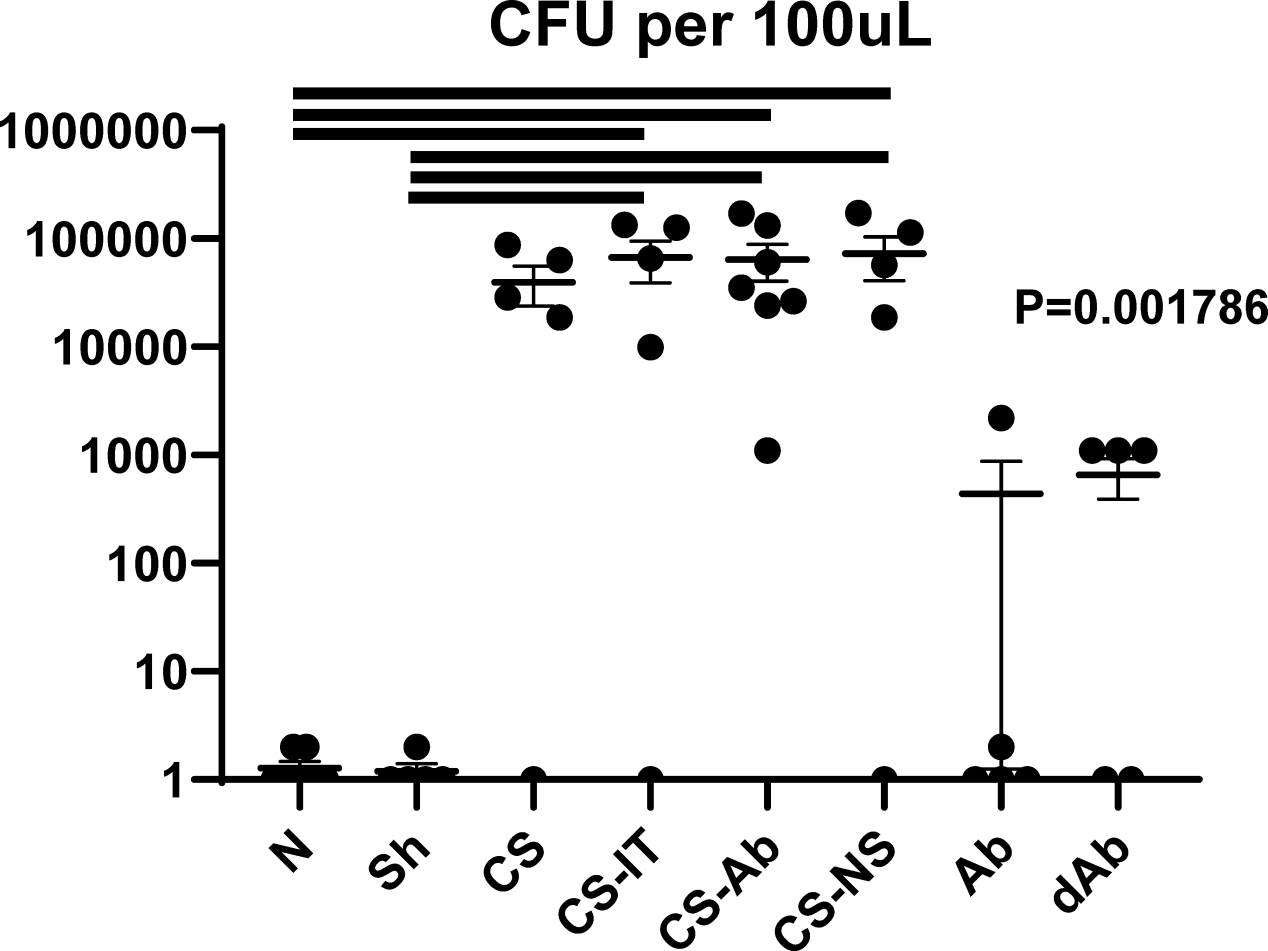
Intraperitoneal bacterial burden. Colony forming units per 100μL of peritoneal fluid presented in log scale, naïve mice demonstrated no bacterial burden whereas all CS treated groups demonstrated significantly more CFUs than naïve samples (0.00 N vs. CS 39783.8, p= 0.032, vs. 72753.8 CS-Ab, p=0.049, vs. 67258.8 CS-IT, p=0.044, and vs. 72534.5 CS-NS, p=0.020). No significant difference between number of CFUs identified existed between the 4 CS treated groups (p=0.66).

CS-Ab treated mice were found to have no significant difference from CS-IT controls in colony forming units in the peritoneal fluid 12hrs after septic insult (p = 0.903, **Figure 8**). Peritoneal lavage samples from naïve pups demonstrated no bacterial burden, while all the CS groups exhibited a marked increase in CFUs (0.00 N vs. CS 39783.8, p= 0.032, vs. 72753.8 CS-Ab, p=0.049, vs. 67258.8 CS-IT, p=0.044, and vs. 72534.5 CS-NS, p=0.020). No significant difference in number of CFUs identified existed between the CS, CS-Ab, and CS-IT treated animals (p=0.66, **Figure 8**). Finally, Ab and dAb treated mice both had low levels of intraperitoneal bacteria, significantly less than any CS treated group (440.6 Ab vs. dAb 659.8 vs. 39784 CS; p=0.013).

## 4 Discussion

This investigation demonstrated intraperitoneal blockade of the HVEM:LIGHT signaling axis resulted in a rapid survival deficit for neonatal mice facing intraabdominal septic challenge. Histologically, this survival deficit was associated with preserved lung histology, thymic karyorrhexis similar to mice who underwent isolated CS treatment, increased thymic cortical HVEM expression, and moderate renal tubular vacuolar degeneration indicative of renal injury. Bacterial burden was not significantly affected by antibody treatment. HVEM:LIGHT signaling blockade produced reduced heart rate and stroke volume, with increases in ejection fraction and fractional shortening, ultimately producing significantly reduced cardiac output compared to sham and CS-IT pups. In total, these results demonstrate checkpoint regulator HVEM is integral to the neonatal response to septic challenge. The rapid lethality and mixed picture of histologic evidence of tissue injury implicate systemic cardiovascular collapse as the cause of death. However, the mechanistic link between HVEM blockade and this cardiac dysfunction was less clear in our investigation. The possibility remains that the observed cardiac dysfunction was due to a robust early cytokine release in the first 18 hours after treatment, given the subtle signals present at 24 hours post treatment, but firm establishment of this causal link will require more investigation.

### 4.1 Survival to septic insult was significantly reduced by HVEM:LIGHT blockade, suggesting an important role for HVEM in septic responses, while resuscitation demonstrated meaningful impact in post-septic survival

It is logical that blockade of the HVEM:LIGHT binding domain results in significantly worse survival to sepsis as the HVEM:LIGHT interaction produces a powerful immune stimulatory response (17). Upon ligation with LIGHT, a robust stimulatory signal results as HVEM recruits TRAF2 to its intracellular domain, activating NFκB, and promoting cell survival (43). Without the ability to stimulate, HVEM is still able to bind and interact with inhibitory ligands BTLA and CD160, generating an uninterrupted inhibitory signal and preventing appropriate immune mobilization (20). Death from isolated blockade of the LIGHT binding domain of HVEM suggests that HVEM triggered immune stimulation is a key component of the immediate neonatal response to sepsis. In this study, death after anti-HVEM antibody administration in sepsis was rapid, implicating more acute phenomena such as cardiovascular collapse or hyperinflammatory cytokine storm. Early mortality in sepsis is almost universally attributed to cardiac dysfunction, in both adults and neonates (9, 44).

Independent delivery of anti-HVEM in the absence of a septic stimuli did not produce lethality, indicating signaling via HVEM:LIGHT is not essential for survival, growth and development in the absence of immune stimulation. CS-IT samples survived significantly better than pups treated with CS alone, and postulate this may be a resuscitative effect of the 20μL of IP fluid mice received with isotype or antibody administration. While this volume is small enough to be negligible in many cases, neonatal study subjects weighed an average of 3.5 grams, making this volume a substantial percentage of their body weight. This was tested by administering CS with an equal volume of 0.9% saline (20μL), and this resuscitation did improve neonatal survival compared to CS alone (14/19 CS-NS vs. 13/28 CS, p=0.037), and more closely recapitulated the isotype groups survival. Knowing this, the survival deficit generated by HVEM:LIGHT blockade, despite the inevitable delivery of this same resuscitation volume, is even more impressive.

### 4.2 HVEM:LIGHT blockade protected normal lung development during septic insult, suggesting a unique and specific role for HVEM signaling in indirect acute lung injury of sepsis

Histologically, HVEM blockade was protective of normal lung development during sepsis, producing morphology most similar to naïve controls despite associated lethality. In contrast, CS-IT lung samples demonstrated histology most similar to the CS alone group despite their robust survival. The low MCL observed in CS-Ab reflects appropriate alveolar remodeling known to occur during the neonatal period in lung development (34). This lung protective effect mirrors results published by Cheng et al. using an adult model of indirect acute lung injury in which intratracheal HVEM siRNA administration was protective of normal lung histology (30). However, in that case, with a more localized delivery of HVEM blockade, a transient survival benefit was derived in the 48hrs following the single intratracheal administration. Septic patients are known to suffer lung injury even in sepsis driven by a compartment outside of the thorax, though this is particularly true in neonates (35). This demonstration, coupled with the feasibility of local intratracheal instillation provides a potentially viable therapeutic strategy to protect neonates from lung injury associated with sepsis, warranting more extensive, focused investigation in the future (30). Additionally, lung protection in the setting of increased mortality suggests the cause of death in these neonates is from an alternate source of septic lethality, such as cardiac dysfunction. One limitation of this study is that neonatal mouse size prohibited use of physiologic measures of impact of this lung injury such as measuring oxygen or CO_2_ exchange, which would characterize the impact of these histologic changes more clearly.

### 4.3 Septic challenge induced signs of AKI and steroid stress within the thymus is not altered by HVEM:LIGHT blockade, despite apparent changes in HVEM expression in the thymic cortex after sepsis

Prior studies of tissue injury in murine sepsis have illustrated lymphoid tissues are particularly susceptible to steroid stress after septic challenge with profound lymphoid organ apoptosis (35, 45). Analysis of H&E-stained thymic samples demonstrated karyorrhexis induced by the CS model, similar in all groups receiving CS, regardless of Ab or IT treatment. These results were confirmed by active caspase 3 staining which suggested increased apoptosis in CS samples with no change in apoptotic extent with antibody administration. This indicates that HVEM:LIGHT blockade does not affect steroid induced injury to lymphoid organs after sepsis, nor does fluid resuscitation. The increased thymic cortical HVEM staining seen in CS-Ab samples suggests HVEM blockade and sepsis do affect the HVEM expression, though the exact extent is not clear.

Acute kidney injury (AKI) is the second most common organ injured as a result of sepsis, behind lung, in adult patients (35). While aging is a major risk factor for severity of kidney injury or failure after sepsis, septic patients of all ages are at risk of AKI, and AKI has an additive negative effect portending a 70% risk of mortality (35, 37). Renal histology showed increased vacuolar degeneration in the tubular cells after sepsis (CS), which was more pronounced in CS-Ab treated mice along with tubular epithelial swelling, both common histologic markers of AKI. Further indications of AKI in septic pups was demonstrated by western blotting, where NGal expression, a biomarker of acute kidney injury, was increased after CS but not significantly altered by resuscitation or CS-Ab.

### 4.4 Significant cardiac dysfunction including significant reduction in cardiac output after CS-Ab treatment accounts for survival deficit

Septic cardiac dysfunction is a well-documented cause of acute mortality after sepsis. Typically occurring with more rapidity than multi-system organ failure, a great deal of research into the central mechanism of this sepsis-associated cardiac dysfunction has failed to identify a specific cause (9). Previously, the role for circulating DAMPs, cytokines, oxidative stress, calcium homeostasis and alterations in nitric oxide production have all been described to contribute to septic cardiac dysfunction (44, 46, 47). Alterations in surface innate immune receptors on cardiomyocytes, including Toll-like receptors, have also been associated with septic cardiac dysfunction, however, the roll of checkpoint regulators such as HVEM in this process have been less clearly defined (46). This study demonstrates that HVEM:LIGHT blockade results in significant cardiac dysfunction in neonates after sepsis, with alterations in nearly every measured parameter of cardiac activity. Blockade resulted in reduced heart rate and stroke volume, suggesting reduction in the necessary compensatory cardiac response to septic hypovolemia. HVEM:LIGHT blockade also increased ejection fraction and fractional shortening, indicating higher strain after sepsis. Finally, blockade produced a significant reduction in cardiac output compared to both Sham and CS-IT treated pups, providing an explanation for the disparate mortality between treatment groups. Interestingly, LVEDV was nearly identical for sham animals and those treated with CS-IT, where sepsis would be expected to result in a hypovolemic shock and reduced LVEDV, arguing that CS-IT survival differed from CS-Ab treatment from a resuscitative benefit.

HVEM is expressed on cardiomyocytes, though no specific role for its presence has been previously defined (19). Looking at mechanistic causes of septic cardiac dysfunction, many of the same cytokines noted to be alternatively produced in neonates after sepsis produce changes in cardiomyocytes that precipitate the cardiac dysfunction. Increases in TNF-α lead to myocyte apoptosis, while increased IL-1, IL-8 and TNF-α increase nitric oxide production which causes DNA damage and ATP depletion which occurs through a decrease in fatty acid and glucose oxidation in the cardiomyocytes. Finally, the increase in NFκB, the direct downstream result of HVEM signaling, impairs beta adrenergic signaling which also leads to apoptosis (9, 11, 44). Given the rapid nature of the mortality noted in the HVEM:LIGHT blockade, peaking between 18 and 24 hrs post insult, cardiac dysfunction is a fitting explanation of mortality as it is classically acute in nature and poorly tolerated, however, this study is limited in its single timepoint examination and expansion to additional timepoint would better illuminate this (9).

### 4.5 Inflammatory cytokine expression was altered by induction of sepsis, and often improved with resuscitation, however HVEM:LIGHT blockade did not alter this at the 24 hr time point

Exploration of inflammatory cytokines at 24 hrs post treatment in the plasma, lung, kidney and thymus, common sites of immune injury, demonstrated several interesting signals. Not surprisingly, in response to CS there was a consistent and marked rise in a number of the pro-inflammatory cytokines in the blood of the neonatal pups, with some echo at the organ level within the kidney and lungs. These tissue specific inflammatory cytokine changes mirror those typically encountered following experimental infectious challenge in many other neonatal and adult sepsis models, and in septic patients (25, 26). Throughout all explored tissues, sepsis increased pro-inflammatory cytokines like TNFα, MCP-1, IL-10 and IL-6 compared to sham animals, regardless of HVEM:LIGHT blockade. However, this effect was often lost with the addition of the equal volume normal saline resuscitation (CS-NS), a clear depiction of the benefit of fluid resuscitation in septic shock. Interestingly, in multiple areas CS-IT treatment similarly resulted in a loss of this significant pro-inflammatory cytokine increase, speaking to the resuscitative benefit of the volume administered with antibody blockade. Further, in the kidney, TNFα increased compared to sham animals only in CS and CS-Ab pups, the highest mortality groups, and those with the most significant evidence of histologic kidney injury.

While HVEM:LIGHT blockade had both negative effects on the CS neonates cardiac function and overall mortality, and improvement in lung injury at 24 hours, this would appear to be generally independent of alteration in the systemic or local organ pro-inflammatory cytokine milieu. On its face, this does not support a role for HVEM:LIGHT induced cytokine signaling as a basis for these morbid changes, and likely speaks to another aspect of HVEM:LIGHT signaling. As previous works have described, HVEM signaling is complex with multiple possible outcomes of ligation depending on the available domains ligated, so it is possible the mortality from HVEM:LIGHT blockade is driven by the preponderance of HVEM:BTLA signaling, however this too ultimately functions via changes in inflammatory cytokines (17, 48). That said, the key limitation to this cytokine exploration is the assessment at a singular acute time point. Given the mortality occurs beginning around 18 hrs after treatment, it is possible that cytokine fluctuations do occur but are transient, and resolving by the 24 hr mark explored here. Also, that anti-body blockade could not alter the CS induced increase in neonatal thymic apoptosis or cause thymic tissue cytokine changes is not totally surprising as this has been documented to be the result of altered steroid levels and largely independent of changes in TNF family members like, TNF and FasL (45). Irrespective, while we cannot completely rule out the role of HVEM:LIGHT driven pro-inflammatory mediator production, locally or systemically, to account for the changes in morbidity and mortality reported here, it is likely that another more tissue specific aspect of HVEM:LIGHT signaling is needed to explain it and will be the basis for future studies.

### 4.6 Future directions

Together, our findings of increased lethality, lung protection, stable thymic apoptosis, acute kidney injury, cytokine alterations, and cardiac dysfunction provide compelling evidence for HVEM’s role in neonatal immune responses to septic challenge. The complexity of HVEM, with its multitude of ligands, and its ability to interact with multiple stimuli simultaneously, serves as both a limitation and a tool. It enables tissue and environmentally targeted outcomes but demands meticulous and extensive interrogation to clarify its multitude of signaling outcomes. The developing neonatal immune system is transitioning from the in-utero environments necessitation of tolerance to a world of aggressive and virulent pathogens, and this ontogeny adds an additional layer of complexity to understanding HVEM’s importance fully (49). While checkpoint regulators such as HVEM are often characterized to act in influencing adaptive responses, the clear effect of HVEM:LIGHT blockade in the neonatal acute death from sepsis hints at a role for regulators such as HVEM in guiding innate responses as well. Ultimately this exploration demonstrates that cardiovascular collapse produces this acute mortality with HVEM:LIGHT blockade, and while other organ systems demonstrated impacts from sepsis consistent with prior research, little else demonstrated the impact of the cardiac dysfunction.

Future experiments are needed to further clarify the specific mechanisms underlying the role HVEM signaling plays in the septic immune response of neonates. The lung protective effect of HVEM:LIGHT blockade, specifically the option of localized administration of anti-HVEM as a potential therapeutic avenue for indirect septic lung injury, requires investigation. While thymic steroid stress was noted at the 24 hr time point, future investigation should explore these impacts at additional time intervals to better identify the impact of the differential cortical HVEM expression noted after sepsis. Characterization of the impact of AKI should involve correlation of blockade with biomarkers such as Creatinine and Blood Urea Nitrogen (BUN). Finally, further work is necessary to better characterize the tissue specific expression of HVEM and all of its ligands, especially in cardiomyocytes. It will be imperative to continue to work to align the understanding of HVEM signaling with the known neonatal immune activity and development. Correlation of these findings with appropriate biomarkers to target potential future therapeutics holds promise of harnessing HVEM to minimize the mortal risks of cardiac dysfunction, lung and/or kidney injury in response to septic challenges.

## Data availability statement

The original contributions presented in the study are included in the article/**Supplementary Material**. Further inquiries can be directed to the corresponding author.

## Ethics statement

All protocols were conducted according to the National Institutes of Health guide for animal care and use and were approved by the Lifespan-Rhode Island Hospital Institutional animal care and use committee (approval number: 0054-18 and 5054-21). The study was conducted in accordance with the local legislation and institutional requirements.

## Author contributions

MW: Writing – review & editing, Writing – original draft, Visualization, Validation, Software, Project administration, Methodology, Investigation, Formal analysis, Data curation, Conceptualization. N-LD: Writing – review & editing, Writing – original draft, Software, Methodology, Investigation, Formal analysis, Data curation. JJ: Writing – review & editing, Methodology, Investigation, Formal analysis. MP: Writing – review & editing, Writing – original draft, Methodology, Investigation, Formal analysis. C-SC: Methodology, Investigation, Writing – review & editing, Supervision. PW: Writing – review & editing, Supervision, Project administration, Methodology, Investigation, Formal analysis. AA: Writing – review & editing, Writing – original draft, Supervision, Resources, Project administration, Funding acquisition, Conceptualization.
