# Supplementary material for: Herpes virus entry mediator signaling blockade produces mortality in neonatal sepsis through induced cardiac dysfunction
Source: Front Immunol. 2024 May 7;15:1365174. doi: 10.3389/fimmu.2024.1365174 (PMC11106455; doi:10.3389/fimmu.2024.1365174)
Supplement: Supplementary file 1 [file DataSheet_1.pdf]

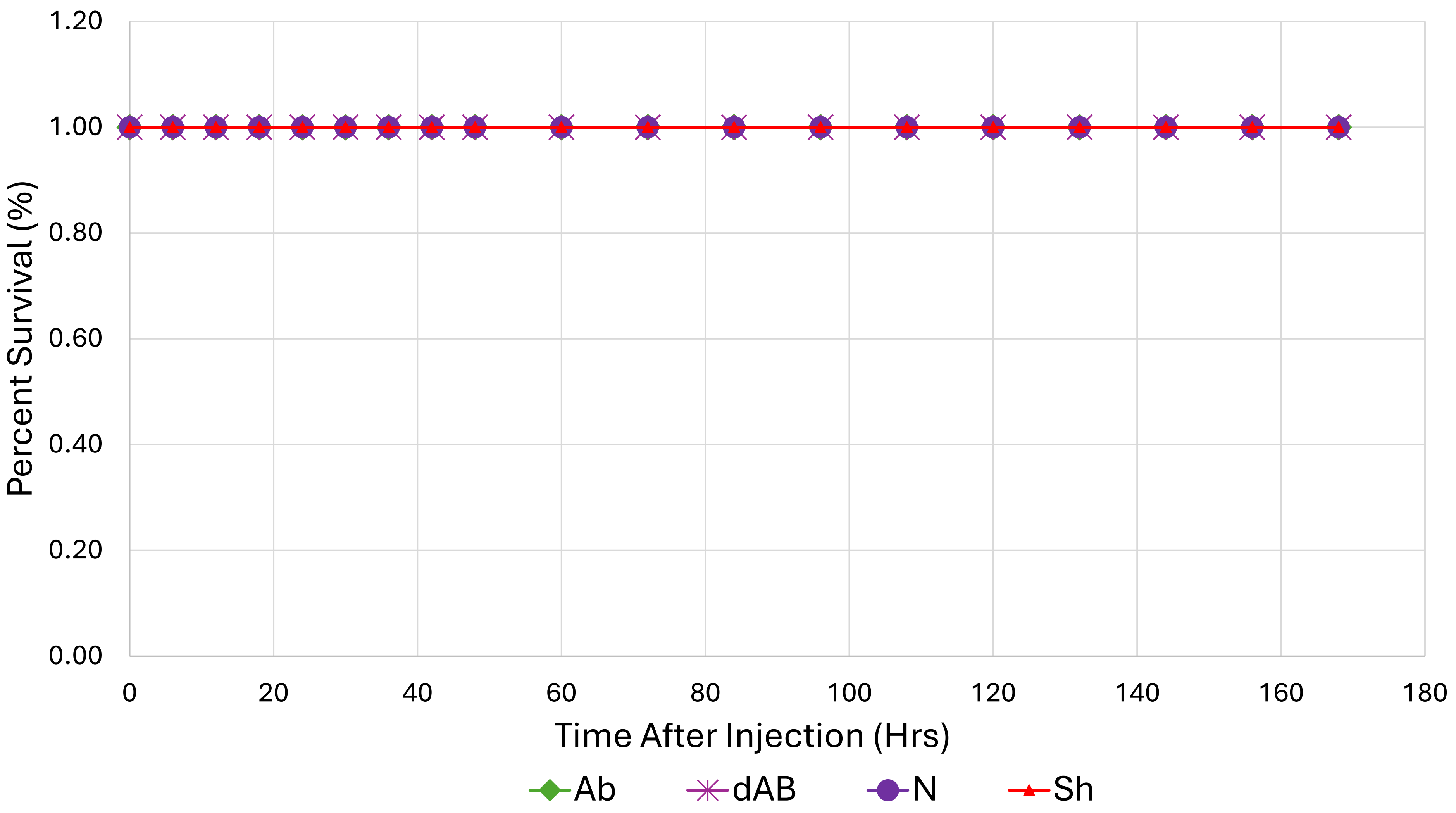

Supplemental Figure 1: Treatment with anti-HVEM antibody alone, or diluted Anti-HVEM in normal saline both produced no mortality, nor was any mortality associated with Naïve mice or Sham intraperitoneal injections (4/4 N, Sh, Ab, dAb); significance * $p<0.05$
