# Supplementary material for: Herpes virus entry mediator signaling blockade produces mortality in neonatal sepsis through induced cardiac dysfunction
Source: Front Immunol. 2024 May 7;15:1365174. doi: 10.3389/fimmu.2024.1365174 (PMC11106455; doi:10.3389/fimmu.2024.1365174)
Supplement: Supplementary file 2 [file DataSheet_2.pdf]

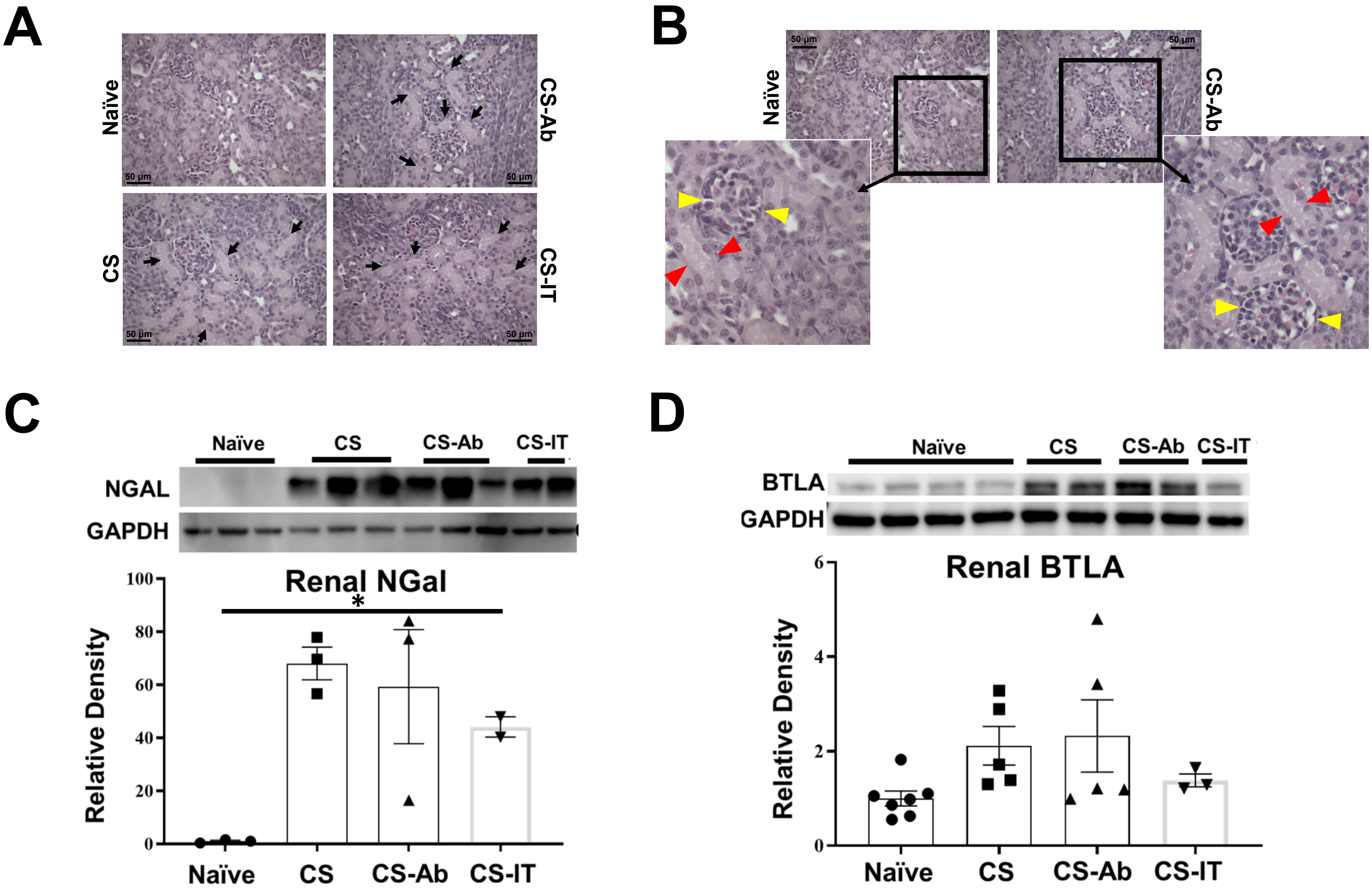

Supplemental Figure 2: Histologic evidence of moderate tubular vacuolization within renal samples after CS, CS-Ab and CS-IT, and renal expression of NGal and BTLA. (**A**) H&E-stained kidney samples harvested from Naïve, CS, CS-IT and CS-Ab treated pups 12 hrs after IP injection depicted at 40x magnification, collected from similar regions, demonstrating expected apical vacuolization in N samples, mild apical tubular vacuolization in 3 of 4 CS samples, while CS-IT samples demonstrated variable findings, most consistently mild partial tubular vacuolization (shown), and 4 of 4 CS-Ab samples with diffuse tubular vacuolization. Black arrows indicate vacuoles. (**B**) Focused sections of N and CS-Ab samples taken at 100x magnification with red arrows denoting normal apical distribution of tubular vacuoles in the naïve sample, and the vacuolar degeneration seen in the antibody treated mice, yellow arrows indicate a normal tubule in the naïve sample and the tubular epithelial swelling noted in the antibody treated mice. (**C**) NGal expression was induced by sepsis, and not significantly affected by antibody administration. Western blots of renal protein samples from Naïve, CS, CS-IT and CS-Ab treated pups 12hrs after IP injection (N=5-7/group; data is shown as histogram with mean +/- SEM; significance * $p<0.05$) stained for NGal and BTLA expression. NGal expression is induced by the administration of CS and maintained in both the CS-Ab and CS-IT groups. (**D**) BTLA expression patterns more closely mirror survival outcomes, however, no changes were significant compared to baseline Naïve samples.
