# Supplementary material for: Herpes virus entry mediator signaling blockade produces mortality in neonatal sepsis through induced cardiac dysfunction
Source: Front Immunol. 2024 May 7;15:1365174. doi: 10.3389/fimmu.2024.1365174 (PMC11106455; doi:10.3389/fimmu.2024.1365174)
Supplement: Supplementary file 7 [file DataSheet_7.pdf]

Thymus

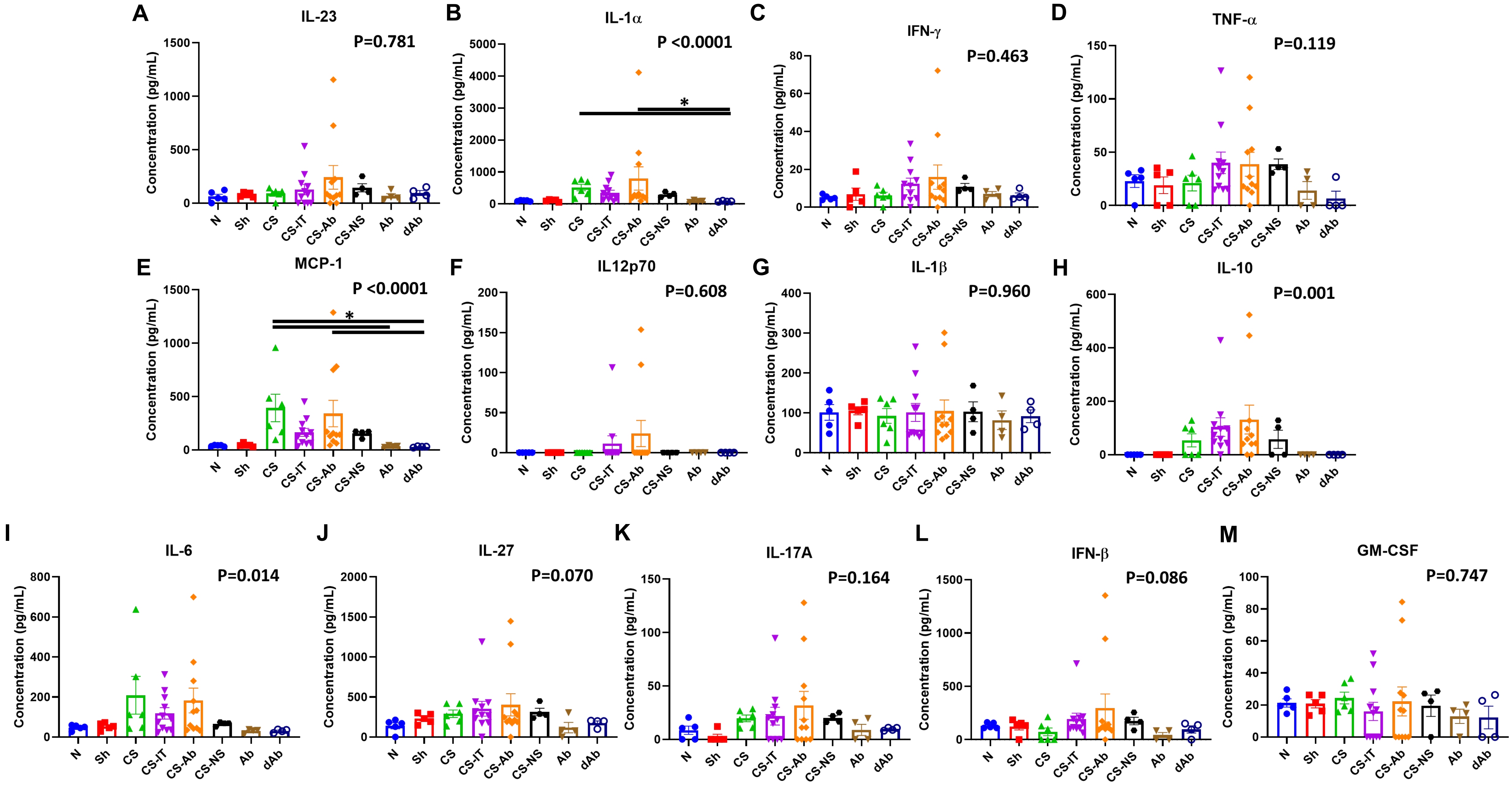

Supplemental Figure 7: Thymus inflammatory cytokine levels 24 hrs after treatment. **(A)** IL-23 **(B)** IL-1α **(C)** IFN-γ **(D)** TNF-α **(E)** MCP-1 **(F)** IL12p70 **(G)** IL-1β **(H)** IL-10 **(I)** IL-6 **(J)** IL-27 **(K)** IL-17A **(L)** IFN-β **(M)**GM-CSF **(A-M)** Summary graphs show data dot plot and mean +/- SEM all after N, Sh, CS, CS-IT, CS-Ab, CS-NS, Ab, and dAb treatments, * significant differences between specific groups (bar) delineated relative to Bonferroni corrected "P" value
